# Optimization of Fillers in Resin-Based Friction Materials and Analysis of Wear Mechanism Through Orthogonal Design

**DOI:** 10.3390/ma19183898

**Published:** 2026-09-13

**Authors:** Lirong Huang, Minjie Huang, Shun Ye, Yuhang Chen, Kai Ming, Junhua Du, Zhigang Liu

**Affiliations:** 1 School of Mechanical and Electrical Engineering, Jiangxi University of Science and Technology, Ganzhou 341000, China; 2 China Copper Huazhong Copper Co., Ltd., Huangshi 435004, China; 3 Jiangxi Huawu Brake Co., Ltd., Fengcheng 331100, China

**Keywords:** resin-based friction materials, wind turbine braking, fly ash cenospheres, tribological performance, eco-friendly brake materials

## Abstract

**Highlights:**

- Eco-friendly ternary filler system successfully replaces toxic antimony additives, utilizing industrial byproducts (fly ash microspheres) alongside MoS_2_ and talc to create a sustainable friction material.
- Optimal formulation (6 wt% MoS_2_, 6 wt% talc, 4 wt% fly ash) achieves excellent tribological performance, with a low wear rate of 0.7 × 10^−7^ cm^3^/(N·m) and 88% friction recovery at 350 °C.
- The complementary functional roles of fillers comprehensively enhance material properties: the talc-FAHMs structural package (combining plate-like talc and rigid spherical fly ash) collectively improves mechanical strength (hardness, compressive strength, and shear strength), while MoS_2_ stabilizes friction performance under thermal cycling.
- Study provides a practical and sustainable design strategy for high-performance brake materials in wind turbine applications, supporting green manufacturing and the use of industrial solid waste.

**Abstract:**

The inherent toxicity of antimony-based fillers in conventional resin-based friction materials necessitates the exploration of sustainable substitutes for wind turbine braking applications. This study therefore aims to formulate a high-performance, environmentally benign alternative using a ternary filler system comprising fly ash microspheres (industrial solid waste), molybdenum disulfide (MoS_2_), and talc. To optimize the filler proportions, an L9(3^4^) orthogonal array design was implemented, systematically assessing the influence of each component on the friction coefficient, wear rate, and mechanical integrity. Dry sliding wear tests were conducted against an HT250 cast iron disc to simulate operational conditions. The experimental results identify the optimal composition (T3: 6 wt% MoS_2_, 6 wt% talc, 4 wt% fly ash) as achieving a low wear rate of 0.7 × 10^−7^ cm^3^/(N·m) at 350 °C, alongside an 88% recovery rate for the friction coefficient, with these key tribological metrics proving directly comparable to those of traditional antimony-containing formulations. The underlying mechanism for this improved performance lies in the synergistic interaction of the three fillers, which facilitates the development of a stable and cohesive tribofilm on the contact surface. Furthermore, analysis confirms that abrasive wear remains the prevailing material removal mechanism, thereby validating the practical feasibility of this newly developed, non-toxic composite material.

## 1. Introduction

In the pursuit of sustainable engineering and environmentally conscious manufacturing, there is a growing imperative to develop high-performance materials that align with global carbon-reduction goals [1,2,3,4]. Friction materials, especially for braking and power transmission systems, are required to exhibit exceptional thermal stability, consistent friction, low wear, and long service life [5,6]. Resin-based composites are widely utilized in this domain due to their ease of processing, cost effectiveness, and formulation flexibility [7,8,9,10,11,12].

In addition to resin-based composites, friction materials for braking applications are also classified into metal-based and ceramic-based categories. Metal-based composites, typically using copper, iron, or steel matrices, offer excellent thermal conductivity, high-temperature strength, and resistance to thermal fatigue, making them suitable for heavy-duty and high-speed braking conditions, such as in aircraft and high-speed trains [13,14]. However, they suffer from high density, limited compressibility, susceptibility to corrosion, and potential environmental concerns related to metallic wear debris [15]. Ceramic-based friction materials, on the other hand, exhibit outstanding high-temperature stability, low density, and good chemical inertness, which are advantageous for extreme thermal environments [16]. Their major drawbacks include intrinsic brittleness, poor machinability, and high manufacturing costs, which restrict their widespread application in large-scale or cost-sensitive industrial components [17]. In contrast, resin-based composites strike a balanced trade-off among processing ease, design flexibility, cost-effectiveness, and adequate tribological performance, which underpins their dominant position in applications such as automotive and wind turbine braking systems [7,8,9,10,11,12].

However, many conventional formulations still rely on toxic fillers such as antimony sulfide, posing environmental and health risks during use and disposal. These materials typically consist of binders, reinforcing fibers, friction modifiers, and fillers [18,19]. Among these, fillers critically determine overall performance. Functional fillers (e.g., MoS_2_, Sb_2_S_3_, talc) enhance friction stability and reduce wear, while structural fillers act primarily as extenders [20,21]. Previous research has extensively explored single additives or binary systems like MoS_2_/Sb_2_S_3_, confirming their benefits in friction reduction and wear resistance [22,23,24]. Optimal loading levels (e.g., 5 wt% Sb_2_O_3_) and more complex ternary systems (e.g., MoS_2_/Bi_2_S_3_/Sb_2_O_3_) have also been identified for performance enhancement [25,26]. Nevertheless, the use of antimony-based compounds remains a major drawback, as they can release inhalable toxic particulate matter (PM) and leachable heavy metal ions, posing risks to ecosystem and human health during wear [27,28,29], conflicting with the demand for eco-friendly solutions.

Our prior work demonstrated that rare-earth oxides (La_2_O_3_, CeO_2_) improve thermal stability and wear resistance by facilitating the formation of a robust, adherent tribofilm [30]. However, that study did not address the well-documented inherent toxicity and associated regulatory and environmental concerns of conventional antimony-based fillers (e.g., Sb_2_S_3_).

For wind turbine brake applications, the target performance criteria include a stable dynamic friction coefficient in the range of 0.30–0.45, a low wear rate to ensure long service life, compressive strength exceeding 90 MPa, and excellent thermal recovery after high-temperature braking events. Prior literature provides useful benchmarks: commercial wind turbine brake pads typically specify dynamic friction coefficients of 0.35–0.45 and abrasion values ≤ 0.02 mm/km. SiO_2_-modified resin-based materials have shown wear rates of 0.22–0.38 × 10^−7^ cm^3^/(N·m), while copper slag-based non-antimony systems exhibit wear rates of 1.2–1.8 × 10^−7^ cm^3^/(N·m) [27]. Fe-Cu-based sintered pads for wind turbines achieved a wear resistance of 4.7 × 10^−8^ g/Nm at a friction coefficient of 0.4, and Cu-based functionally gradient composites reported wear rates of 2.013 × 10^−7^ g·N^−1^·m^−1^ with a friction coefficient of 0.215.

To bridge the critical gap between performance requirements and environmental safety, the present study introduces a novel antimony-free ternary filler system composed of fly ash hollow microspheres (FAHMs), molybdenum disulfide (MoS_2_), and talc. In this system, FAHMs serve as sustainable, waste-derived structural reinforcements; MoS_2_ provides solid-lubrication functionality; and talc contributes to thermal buffering and interfacial cohesion. These constituents are deliberately selected to achieve synergistic improvements in lubrication, load-bearing capacity, and thermal resilience, without compromising environmental safety or regulatory compliance. It should be noted that the present investigation is confined to the screening of filler formulations and the evaluation of their short-term tribological performance under controlled thermal cycling. For wind turbine braking systems, which operate outdoors and undergo repeated braking events, long-term environmental degradation (e.g., moisture, UV, thermal ageing) and mechanical fatigue are also critical for service life. These aspects are beyond the scope of this initial optimization study but will be systematically addressed in subsequent work. The overarching aim of this work is therefore to develop a material that meets or exceeds the above benchmarks while eliminating toxic antimony-based additives.

## 2. Materials and Methods

### 2.1. Experimental Materials

All fillers and functional additives used in this study were obtained from certified suppliers based in China. A complete list of the materials, including grades, supplier information and primary role, is provided in Table 1.

The selection of the weight fraction ranges for the three key variable fillers (MoS_2_, talc, and FAHMs) was based on literature precedents, preliminary tests, and industrial applicability. For layered MoS_2_, a well-established solid lubricant, the range of 6–10 wt% was chosen to ensure effective lubrication while avoiding excessive compromise of matrix cohesion, aligning with typical loadings in high-performance formulations [22,23]. The plate-like talc content (3–6 wt%) represents a common range for leveraging its dual role as a lamellar lubricant and a structural reinforcer. The FAHMs are classified as Class F fly ash per GB/T 1596-2017 [31], with hollow spherical morphology as specified by the supplier and documented in prior studies [32]. The incorporation level of FAHMs (2–4 wt%) was designed to meaningfully valorize this industrial solid waste without adversely affecting the density and processability of the composite, a strategy supported by prior work on eco-friendly fillers [27,28]. These ranges ensure that the total variable filler content remains within industrially realistic bounds for resin-based friction materials.

### 2.2. Experimental Design

To efficiently screen the main effects of the three key fillers (MoS_2_, talc, and FAHMs), an L_9_(3^4^) orthogonal array was employed, as detailed in Table 2. This design enables a balanced and representative preliminary exploration of the three-factor, three-level system using only nine experimental runs, striking an optimal balance between statistical efficiency and experimental feasibility for initial formulation optimization under resource constraints.

The primary objective of this stage is to identify dominant trends and locate a promising formulation region, rather than to pinpoint a global optimum with high precision. Consequently, the optimal formulation identified via the L_9_(3^4^) array represents the best-performing combination among the nine discrete compositions tested. It establishes a robust performance baseline for this study and serves as a central reference point for future, more refined optimization.

Although it provides unbiased and efficient estimation of main effects, it inherently confounds two-factor and higher-order interactions due to its fractional factorial structure. As a result, synergistic mechanisms among fillers cannot be statistically isolated or quantified within this design alone. Consequently, our discussion of coordination integrates main-effect trends with corroborating microstructural evidence (e.g., dispersion morphology, interfacial bonding, phase distribution), rather than relying on interaction terms from an incomplete statistical model.

In summary, the L_9_(3^4^) orthogonal array employed here serves as an efficient screening tool, successfully identifying a promising baseline formulation (T3) for subsequent development. However, a key limitation of this design is the intrinsic coupling of talc and FAHMs mass fractions, which vary synchronously when MoS_2_ content is held constant. Consequently, this setup does not allow for independent estimation of the main effects or interaction between these two fillers. Any interpretations regarding their individual contributions to mechanical properties are therefore necessarily qualitative and should be understood as combined effects. Decoupling and quantifying their respective roles will require dedicated future studies using full factorial or mixture design approaches.

### 2.3. Specimen Preparation

**Figure 1:**
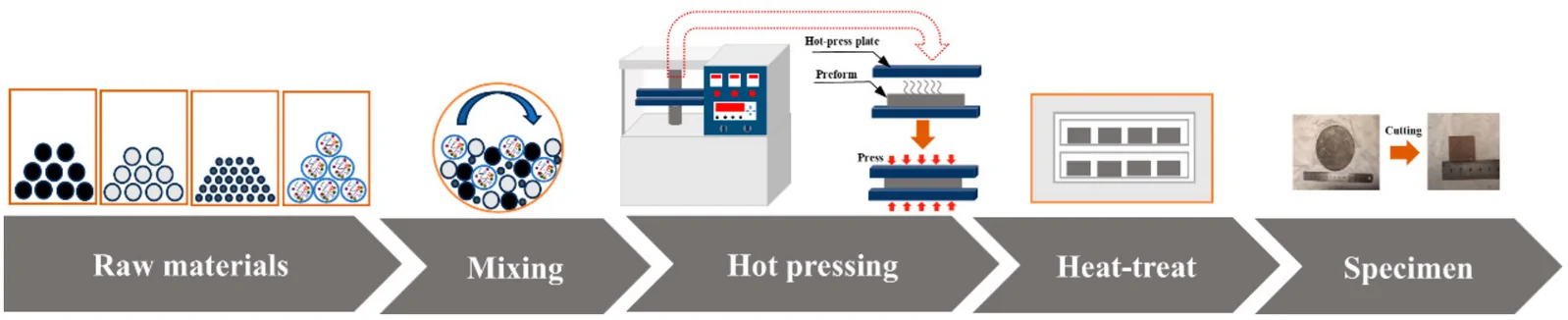
Schematic diagram of the hot pressing process.

The preparation protocol followed a sequential “pre-mixing → blending → molding → post-processing” workflow to ensure compositional homogeneity and microstructural consistency, as illustrated in Figure 1.

First, raw materials for each batch were accurately weighed using an analytical balance (±0.1 mg precision). To prevent fiber agglomeration and promote uniform distribution, aramid fibers were initially pre-dispersed in a JFH-20 high-shear mixer (Zhengzhou Jinhe Powder Technology Co., Ltd, Zhengzhou, China) at 1000 r/min for 5 min.

Subsequently, the remaining constituents were added incrementally, and blending continued for an additional 15 min under the same shear conditions, yielding a homogenized mixture ready for consolidation.

The mixture was then transferred into a JFY60 hot-press mold (Jilin Province Jida Elec.-Mech.Machinery Ltd, Changchun, China) and consolidated under a uniaxial pressure of 15 MPa. This pressure falls within the typical range (10–20 MPa) reported for resin-based brake materials, balancing adequate densification with practical process feasibility [7,30]. Temperature profiles across the mold were precisely controlled at 150 °C (top platen), 160 °C (center cavity), and 150 °C (bottom platen) to ensure uniform heat transfer and complete resin flow. Isothermal curing was maintained for 600 s, allowing the matrix to polymerize into a solid “green” compact.

To achieve full cross-linking and minimize residual thermal stress, the green compacts were post-cured in a forced-convection oven at 180 °C for 12 h, followed by slow furnace-cooling to ambient temperature (25 ± 2 °C). Finally, the fully cured specimens were machined to standardized test dimensions on an MQD3220 precision cutting system (Wuhan Wumao Electromechanical Equipment Co., Ltd, Wuhan, China) maintaining dimensional tolerances within ±0.05 mm.

This well-established preparation protocol, particularly the high-shear blending and controlled hot-press molding, is widely adopted for resin-based composites to ensure a homogeneous distribution of fillers and fibers. The consistent processing conditions provide a reproducible baseline microstructure, which is essential for reliable and comparable tribological evaluation.

### 2.4. Performance Characterization

#### 2.4.1. Mechanical Properties

The shear strength and compressive strength of the composite materials were evaluated using a WAW600KN hydraulic universal testing machine (Guilin Ruite Testing Machine Co., Ltd, Guilin, China) in accordance with GB/T 26739-2011 [33] and GB/T 1041-2008 [34] standards, respectively. Rockwell hardness (HRL) was measured with an XHR-150 plastic Rockwell hardness tester (Shanghai Precision Instrumentation Co., Ltd, Shanghai, China), employing a 6.350 mm diameter steel ball indenter, an initial test force of 98.07 N, and a total test force of 588.4 N, following GB/T 5766-2007 [35]. For each specimen, five independent measurements were taken at different locations, and the average value was reported to ensure data reproducibility and account for potential local inhomogeneity.

#### 2.4.2. Friction and Wear Behavior

For each specimen, two independent measurements were taken at different locations. The mean and standard deviation were calculated, with the mean used as the representative value for analysis.

**Figure 2:**
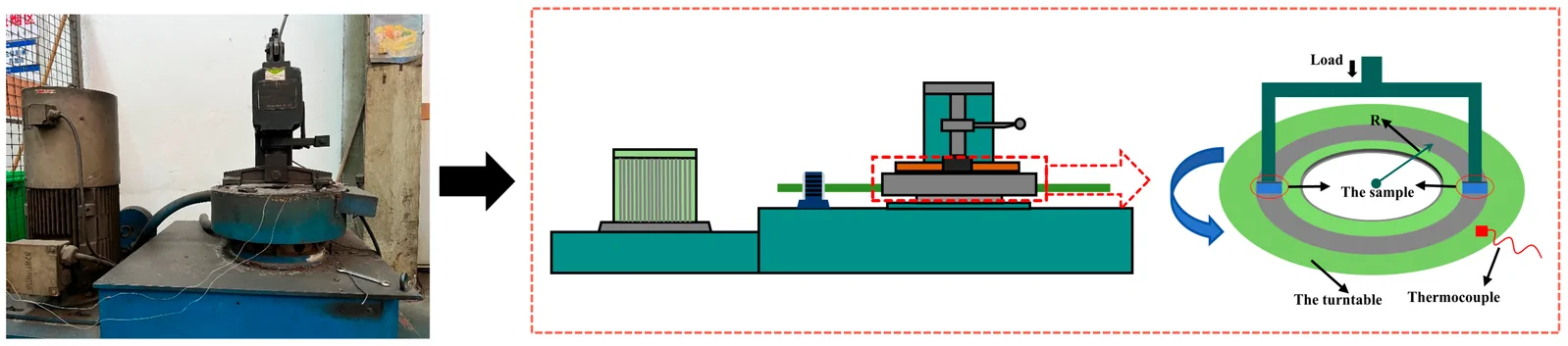
Schematic illustration of the XD-MSM fixed-speed friction and wear tester.

The friction coefficient and wear rate were assessed according to GB5763-2008 [36] using an XD-MSM fixed-speed friction and wear tester (Xianyang Xinyi Friction and Sealing Equipment Co., Ltd, Xiangyang, China); its structural schematic is shown in Figure 2. The specimen dimensions were 25 × 25 mm in contact area with a thickness of 5–7 mm. The counterpart disk was made of pearlitic gray cast iron HT250 (Brinell hardness HB180–220) with a radius of 0.15 m. The specimen is mounted in the upper holder, while the cast iron disc rotates horizontally. The thermocouple is placed approximately 1 mm behind the friction surface to record the bulk temperature. The counter-disc surface was finished to a roughness of Ra ≈ 0.8 μm. The friction force is measured continuously by a load cell integrated into the specimen holder.

The tests were conducted under dry sliding conditions, consistent with the intended application in dry multi-disc brake systems for wind turbine generators. To simulate interfacial conditions during controlled or emergency stops, a constant pressure of 0.98 MPa was applied, which falls within the typical operating range (0.5–1.5 MPa) for such systems. The disk rotated at a steady speed of 490 r/min, corresponding to a sliding speed of approximately 7.7 m/s, representative of relative surface speeds in wind turbine braking. This combination of pressure and sliding speed generates interfacial conditions critical for tribofilm evaluation.

To evaluate thermal stability and recovery, which are key requirements for wind turbine brakes, the tests spanned a temperature range of 100 °C to 350 °C, covering both heating and cooling phases. This range captures the critical transition from stable operation (≈100–200 °C) to the onset of thermal decomposition and potential fade (250–350 °C). The upper limit of 350 °C is relevant for assessing safety margins under severe braking. The reported temperature refers to the uniformly controlled environmental chamber temperature, measured by a calibrated thermocouple (±2 °C accuracy), providing a consistent bulk thermal state for performance evaluation.

Prior to testing, the disk surface was polished with P240 grit sandpaper per JB/T 749-2018 [37] and cleaned with acetone. Each specimen was ground until contact area exceeded 95%. Friction and wear data were recorded at each specified temperature set-point (100, 150, 200, 250, 300, and 350 °C) during both heating and cooling phases, after stabilizing the temperature within ±2 °C for 2 min at each point.

The friction coefficient was calculated automatically by the system software using Equation (1):

(1) $$\mu =\frac{f}{F}$$

Among them, *μ* denotes the coefficient of friction, f represents the average friction force (N), and F denotes the normal pressure (N);

The wear rate of each sample was calculated according to Equation (2) after completing 5000 revolutions at each of the above-mentioned stabilised temperature set-points.

(2) $$W_{r}=\frac{1}{2\pi R}\;\times \;\frac{A}{n}\;\times \;\frac{d_{1}-d_{2}}{f_{m}}$$

In the formula, R denotes the distance between the center of the specimen and the axis of rotation of the disc (m); n represents the total number of revolutions of the disc; A refers to the total area of the specimen’s friction surface (cm^2^); $d_{1}$ and $d_{2}$ indicate the average thickness of the specimen before and after testing (cm), respectively; and $f_{m}$ denotes the total average friction force (N).

Equation (2) is derived from the direct measurements of thickness loss, friction force, and rotational kinematics in our fixed-speed friction tester. It is dimensionally consistent and equivalent in principle to the Archard wear equation $V=K\cdot \frac{F_{N}\cdot L}{H}$, where V is wear volume, $F_{N}$ is normal load, L is sliding distance, and H is hardness, and K is the dimensionless wear coefficient that characterizes the wear resistance of the specific material pair under given tribological conditions. L=2πR, and the average friction force is $f_{m}=\mu \cdot F_{n}$. Equation (2) yields the wear rate in the standard unit of cm^3^/(N·m), aligning with common tribological practice while being tailored to our experimental apparatus.

#### 2.4.3. Microscopic and Structural Analysis

The worn surface morphology and elemental composition were examined using an MLA650F field emission scanning electron microscope (SEM) equipped with an energy-dispersive X-ray spectrometer (EDS) (FEI Company, Hillsboro, OR, USA). Surface topography was quantitatively analyzed with an MT-500 Probe Type Material Surface Abrasion Measuring Instrument (Lanzhou Zhongke Kaihua Science and Technology Development Co., Ltd, Lanzhou, China). The phase structure was characterized using an Empyrean X-ray diffractometer (XRD) (Malvern Panalytical, Almelo, The Netherlands).

While SEM-EDS and XRD provide valuable information on the elemental distribution and crystalline phases present on the worn surfaces, these techniques do not offer definitive identification of the chemical oxidation states of molybdenum species. Surface-sensitive techniques such as X-ray photoelectron spectroscopy (XPS) would be required to conclusively determine the MoS_2_/MoO_3_ ratio and the detailed chemistry of the tribofilm. This limitation is acknowledged, and the interpretations presented herein are accordingly framed with appropriate caution.

## 3. Results

### 3.1. Mechanical Properties

**Figure 3:**
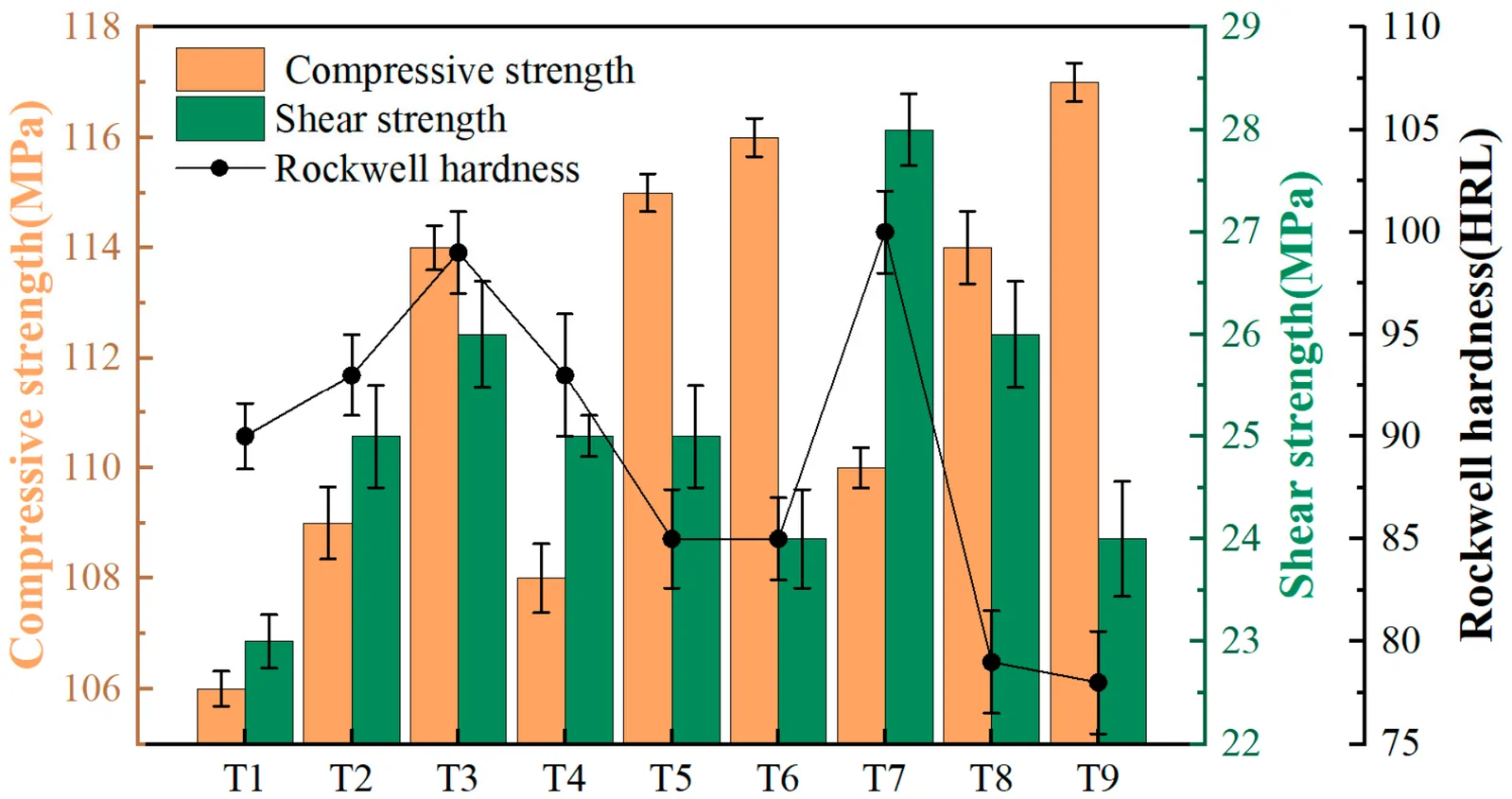
Mechanical properties of materials.

As shown in Figure 3, the mechanical properties of the friction material varied with the combined talc and FAHMs content. The hardness of all specimens ranged from 78 to 100 HRL, with T7 exhibiting the highest value (100 HRL) and T9 the lowest (78 HRL). The compressive strength ranged from 106 to 117 MPa, with T9 reaching the maximum and T1 the minimum. The shear strength ranged from 23 to 28 MPa, with T7 showing the highest value and T1 the lowest. Since talc and FAHMs contents are fully coupled in this L_9_(3^4^) design, the observed variations in these properties cannot be statistically attributed to either filler individually. Nevertheless, higher combined loadings of talc and FAHMs generally improved compressive strength (e.g., T3 and T9) and also tended to raise hardness, while the shear strength varied with the combined filler content. These trends are likely attributable to the complementary reinforcement mechanisms: talc plates provide interlocking and promote stress transfer, crack deflection, and interlaminar cohesion, while spherical FAHMs provide rigid load-bearing sites and act as anchors to enhance interfacial shear resistance; their synergistic effect is likely responsible for the observed changes.

### 3.2. Friction and Wear Properties

The friction and wear data presented below are based on two repeated tests for each formulation. Error bars in the figures represent the standard deviation, reflecting the consistency of the measurements.

#### 3.2.1. Friction Coefficient

**Figure 4:**
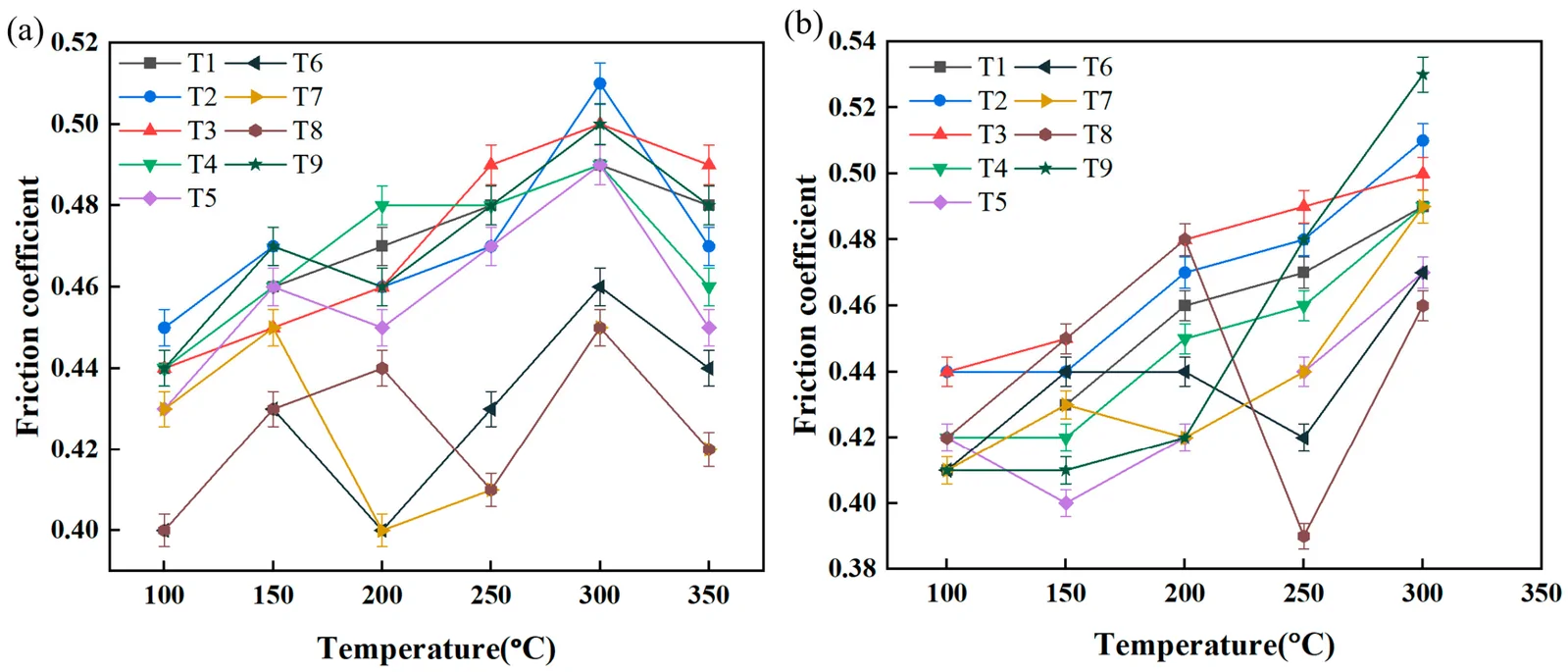
Variation in the friction coefficient of the specimens with temperature: (**a**) heating phase; (**b**) cooling phase.

As shown in Figure 4a, the friction coefficient of most specimens reached a peak at 300 °C, followed by a decrease at 350 °C, indicating the onset of thermal fade. The heating-and-cooling cycle employed here provides a first-order assessment of thermal stability and recovery, which can be regarded as a simplified thermal fatigue indicator. Nevertheless, this test does not fully replicate prolonged mechanical fatigue or environmental ageing; those factors will be investigated in future dedicated studies.

This initial rise is tentatively attributed to the partial oxidation of MoS_2_. While some studies have reported that MoO_3_ can exhibit lubricious properties due to its layered crystal structure with weak van der Waals bonding and can contribute to protective tribofilm formation [38,39], other works suggest that excessive oxidation to MoO_3_ may lead to increased friction and wear under certain conditions. The subsequent decline at 350 °C likely results from the combined effects of lubricant depletion, changes in the MoS_2_/MoO_3_ balance, and the onset of thermal degradation of the phenolic resin matrix. These processes may destabilize the protective tribofilm, leading to increased direct asperity contact [40].

Analysis of the friction-coefficient curves reveals that the T3 specimen displayed the smallest degradation in friction performance within the 300–350 °C range, coupled with a smoother curve, suggesting superior thermal stability. This outstanding stability is quantitatively confirmed by its high friction recovery rate of 88% after cooling. The superior stability of the T3 specimen is directly linked to its optimal filler coordination, which mitigates these degradation mechanisms: FAHMs enhance thermal stability, talc maintains interfacial cohesion, and the balanced MoS_2_ content provides a sustained lubricant reservoir, thereby buffering the friction transition at high temperatures.

**Figure 5:**
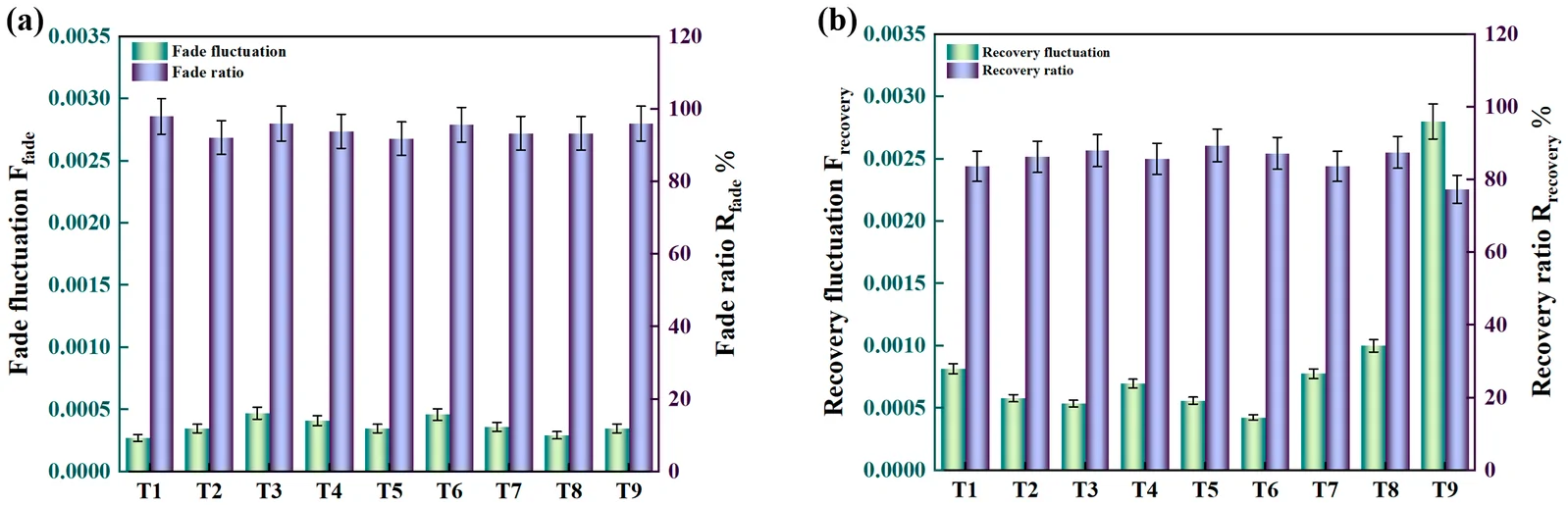
The variance, decay rate, and recovery rate of material’s friction coefficient: (**a**) recession process; (**b**) recovery process.

To better evaluate the stability of the material’s friction coefficient, Equations (3)–(6) were used to calculate the variance, decay rate, and recovery rate of the friction coefficient for each group of specimens [41], with the results presented in Figure 5.

(3) $$F_{\mathrm{fade}}=\sum_{i=1}^{n}{\left({\bar{\mu }}_{f}-\mu_{i}\right)}^{2}/n$$

(4) $$F_{re\mathrm{cov}ery}=\sum_{j=1}^{n}{\left({\bar{\mu }}_{r}-\mu_{j}\right)}^{2}/n$$

In the equation , n is the number of temperature conditions for each test, ${\bar{\mu }}_{f}$ and ${\bar{\mu }}_{r}$ represents the average friction coefficient during the recession and recovery processes, respectively; $\mu_{i}$ and $\mu_{j}$ denotes the friction coefficient at each specific temperature during these processes.

(5) $$R_{fade}=\frac{\mu_{f-350\;{}^{\circ}C}}{\mu_{f-\max}}\times 100\%$$

(6) $$R_{recovery}=\frac{\mu_{r-100\;{}^{\circ}C}}{\mu_{r-\max}}\times 100\%$$

Among them, $\mu_{f-350\;{}^{\circ}C}$ and $\mu_{f-\max}$ represent the friction coefficient at 350 °C and the maximum friction coefficient during the recession phase, respectively; $\mu_{r-100\;{}^{\circ}C}$ and $\mu_{r-\max}$ denote the friction coefficient at 100 °C and the maximum friction coefficient during recovery phase, respectively.

From Figure 5a, the order of thermal recession resistance is T1 > T3 > T9 > T6 > T4 > T7 > T8 > T2 > T5, while the order of stability is T1 > T8 > T5 > T2 > T9 > T7 > T4 > T6 > T3. This indicates that the T1 specimen exhibits the highest resistance to the friction coefficient decline during the recession process, followed by the T3 specimen. From Figure 5b, the order of recovery rate is T5 > T3 > T8 > T6 > T2 > T4 > T7 > T1 > T9, and the order of stability is T6 > T3 > T5 > T2 > T4 > T7 > T1 > T8 > T9. The results show that during the recovery process, the T6 specimen exhibits the best stability, followed by the T3 specimen; however, the recovery rate of the T3 specimen is higher than that of the T6 specimen, indicating that the T3 specimen maintains the most stable friction coefficient overall during the recovery stage. By integrating the results from Figure 5a,b it can be concluded that the T3 specimen demonstrates superior overall performance in terms of the friction coefficient stability and resistance to degradation.

#### 3.2.2. Wear Rate

**Figure 6:**
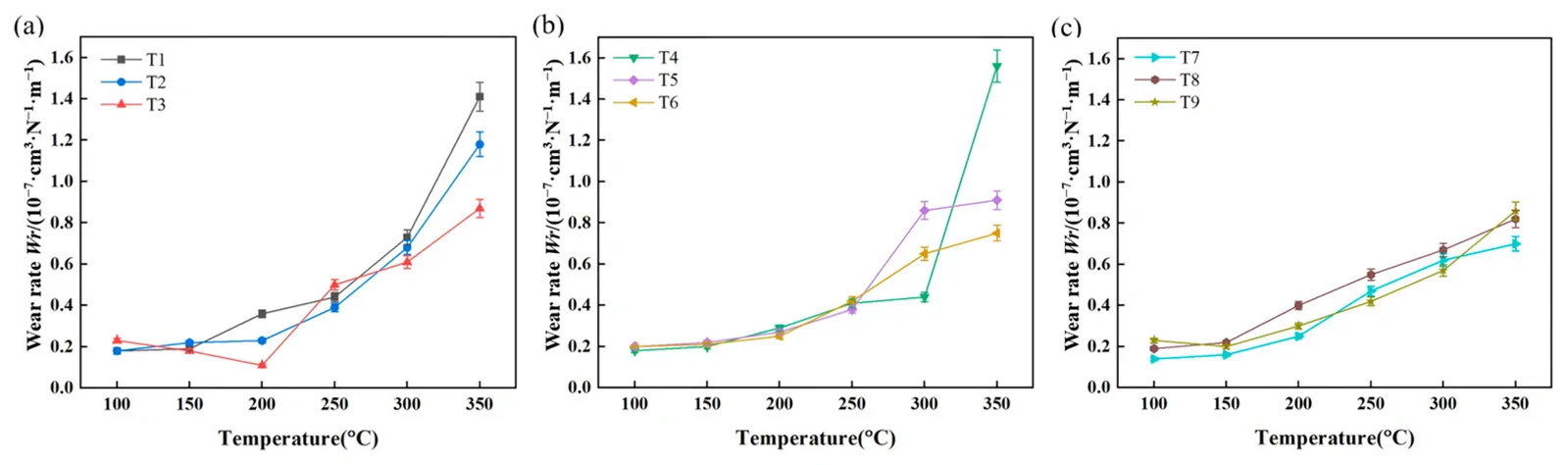
Variation in the wear rates of specimens with temperature: (**a**) wear rate of T1, T2 and T3; (**b**) wear rate of T4, T5 and T6; (**c**) wear rate of T7, T8 and T9.

The wear rates of each group of specimens were measured at different braking temperatures, with the results presented in Figure 6. As shown in Figure 6, the wear rate of all specimens increases with rising temperature. In the low-temperature range of 100–200 °C, the differences among specimens are relatively small. However, as temperature rises further, distinct trends emerge: at 200 °C, the T3 specimen exhibits the lowest wear rate of 0.11 × 10^−7^ cm^3^/(N·m), while in the high-temperature range of 300–350 °C, the wear rates of T1, T2, and T4 increase significantly. Among these, T4 shows the most pronounced increase, reaching a maximum of 1.56 × 10^−7^ cm^3^/(N·m) at 350 °C.

The observed inverse relationship between the friction coefficient (Figure 4) and wear rate (Figure 6) at elevated temperatures (e.g., 350 °C) is a direct consequence of the degradation of the protective tribofilm. As described in Section 3.2.1, the friction coefficient decrease at 350 °C is attributed to the depletion of the MoS_2_ lubricant, its oxidation to abrasive MoO_3_, and the thermal softening of the resin matrix. These processes destabilize the continuous, protective tribofilm. The loss of this tribofilm leads to increased direct asperity contact between the composite and the counterface, resulting in severe abrasive wear and higher material removal rates. Therefore, while the friction coefficient decreases due to reduced interfacial shear strength and lubricant failure, the wear rate increases due to the loss of tribofilm protection and the presence of abrasive oxidation products. This mechanistic linkage clarifies that the tribological behavior under severe conditions is consistent with a transition from a tribofilm-protected, stable regime to a direct-contact, high-wear regime.

Notably, the optimal T3 formulation maintains a low wear rate of 0.7 × 10^−7^ cm^3^/(N·m) even at temperature of 350 °C. This value is significantly lower than those reported for other non-antimony systems utilizing industrial wastes such as copper slag (1.2–1.8 × 10^−7^ cm^3^/(N·m)) [27], and is comparable to some commercial low-metallic formulations [7]. These results demonstrate that the coordination between FAHMs and MoS_2_ in the T3 system not only eliminates toxic fillers but also achieves competitive wear resistance under demanding thermal conditions, a critical advancement in the design of high-performance green friction materials.

### 3.3. Two-Dimensional Contours

**Figure 7:**
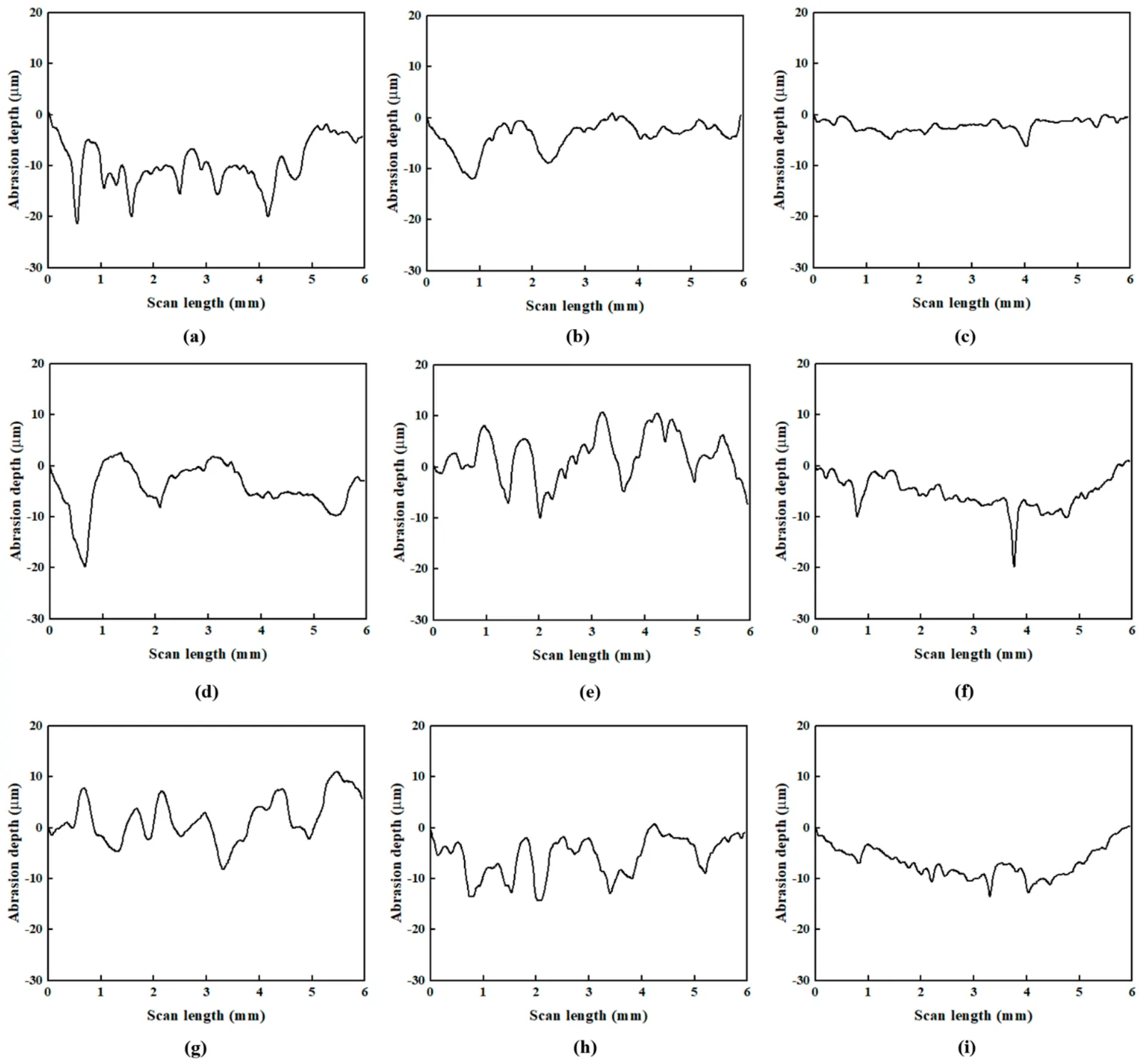
Two-dimensional morphology of the wear surface among the specimens: (**a**) T1; (**b**) T2; (**c**) T3; (**d**) T4; (**e**) T5; (**f**) T6; (**g**) T7; (**h**) T8; (**i**) T9.

Two-dimensional contour analysis was conducted to further investigate the microscopic effects of different fillers on the friction properties of brake materials. Micro-scratch tests were performed on each group of specimens, and the results are presented in Figure 7.

As shown in Figure 7, the T1 specimen exhibits a maximum abrasion depth of −21.307 µm and displays the widest wear groove among all specimens. This indicates that the wear surface of this specimen features deep and wide grooves, reflecting a more severe degree of abrasive wear. Localized surface protrusions are observed on the T7 and T5 specimens, which may result from the embedding of hard particles into the material surface during the friction process. The T6 and T8 specimens exhibit maximum abrasion depths of −19.734 µm and −14.307 µm, respectively, with the T8 specimen showing a higher number of abrasion marks, suggesting relatively more pronounced wear characteristics. Among all specimens, the T3 and T7 specimens show the shallowest abrasion depths, indicating superior surface flatness and reduced wear severity.

### 3.4. Wear Patterns

The wear mechanisms were classified based on distinct morphological features observed in the SEM micrographs, following established tribological interpretations. Adhesive wear was identified by features such as material transfer, tearing, plucking, and the formation of adhered layers or large cavities resulting from localized bonding and subsequent detachment at the interface. Abrasive wear was characterized by parallel scratching, grooving, ploughing, and the presence of micro-cutting chips, resulting from the penetration and sliding of hard asperities or third-body particles. Mixed wear refers to surfaces exhibiting a combination of both adhesive and abrasive features. This classification provides a qualitative framework for linking surface morphology to the dominant wear process.

**Figure 8:**
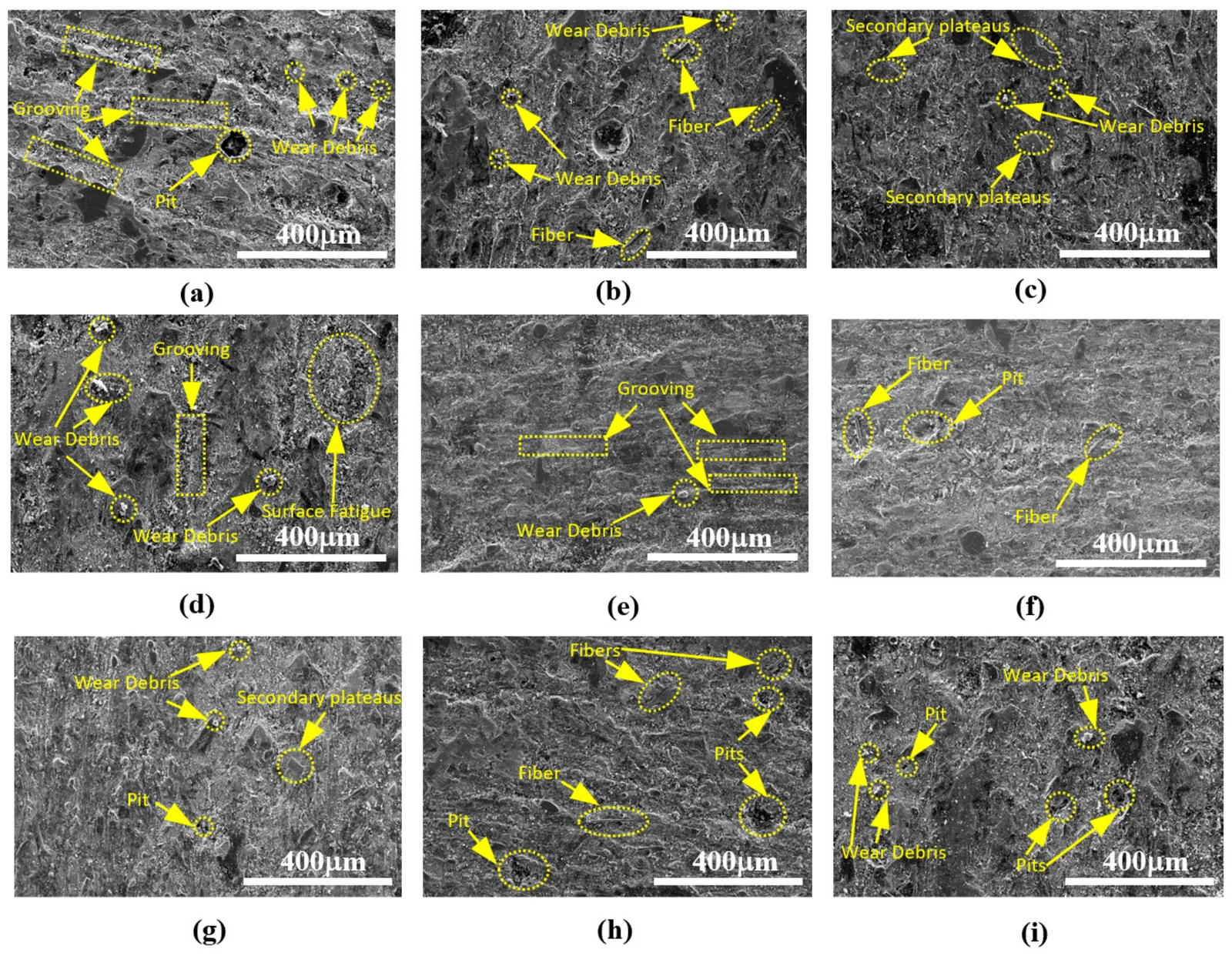
SEM images of wear surface morphology among the specimens: (**a**) T1; (**b**) T2; (**c**) T3; (**d**) T4; (**e**) T5; (**f**) T6; (**g**) T7; (**h**) T8; (**i**) T9.

SEM examination of the worn surfaces (Figure 8) revealed four dominant categories. Adhesive-dominated wear was evident in T1, T4, and T6: T1 (Figure 8a) showed deep furrows, flaking craters, and abundant debris; T4 (Figure 8d) exhibited slight furrowing with plastic deformation and peeled-off debris; and T6 (Figure 8f) displayed the most severe damage, with large adhesive pits and material spallation. Abrasive-dominated wear characterized T2, T5, and T8: T2 (Figure 8b) showed partially exposed fibers and spalling pits, T5 (Figure 8e) exhibited clear abrasion marks and abrasive chips, and T8 (Figure 8h) presented holes and exposed fibers with relatively fewer abrasion marks. Mixed wear was identified in T7 and T9 (Figure 8g,i), where abrasive grooves coexisted with localized material detachment. In contrast, T3 (Figure 8c) displayed extensive, uniformly distributed, and well-adhered tribofilms covering an estimated >80% of the observed surface area, with only minor isolated damage, whereas T1 and T6 exhibited discontinuous tribofilms with coverage below 50% and large areas of direct composite exposure. This semi-quantitative assessment of tribofilm continuity, based on comparative observation across multiple SEM fields, provides a direct morphological correlate to the macroscopic wear rates shown in Figure 6.

These morphological categories should be interpreted within a tribofilm dynamics framework. Where a continuous, adherent tribofilm is present (e.g., T3), surface smoothness and shallow abrasion marks reflect the dominance of tribofilm-mediated shear and compaction, rather than the absence of wear. In this regime, wear debris is rapidly incorporated into a protective third-body layer, and material loss occurs through mild, localized delamination and re-formation of the film. Conversely, where the tribofilm is absent or discontinuous (e.g., T1, T4, T6), deep grooves, spalling pits, and exposed fibers indicate direct wear, with hard asperities and debris ploughing and cutting the matrix and fibers. The distinction between specimens is therefore not merely one of wear intensity, but of fundamentally different surface protection mechanisms.

The uniform, adherent tribofilm on T3 is consistent with mechanisms reported for high-performance systems containing solid lubricants such as Sb_2_S_3_ or graphite [22,26]. However, in those conventional systems, tribofilm stability is often attributed to metallic sulfides. In contrast, the effective tribofilm formation achieved in our antimony-free system demonstrates that the coordination between FAHMs (providing mechanical support and heat dissipation) and MoS_2_ (providing lubrication and shear planes) can replicate this crucial function without toxic additives.

### 3.5. Comparative EDS and XRD Analysis of Optimal and Poor-Performing Specimens

To gain deeper insight into the mechanisms underlying the superior performance of the optimal formulation, a comparative EDS and XRD analysis was conducted on the worn surfaces of the optimal specimen (T3) and a poor-performing specimen (T6). Specimen T6 was chosen as the counterpart due to its high wear rate (Figure 6) and severe surface damage characterized by large pits and semi-exposed fibers (Figure 8f), which starkly contrasts with the uniform tribofilm observed on T3 (Figure 8c).

**Figure 9:**
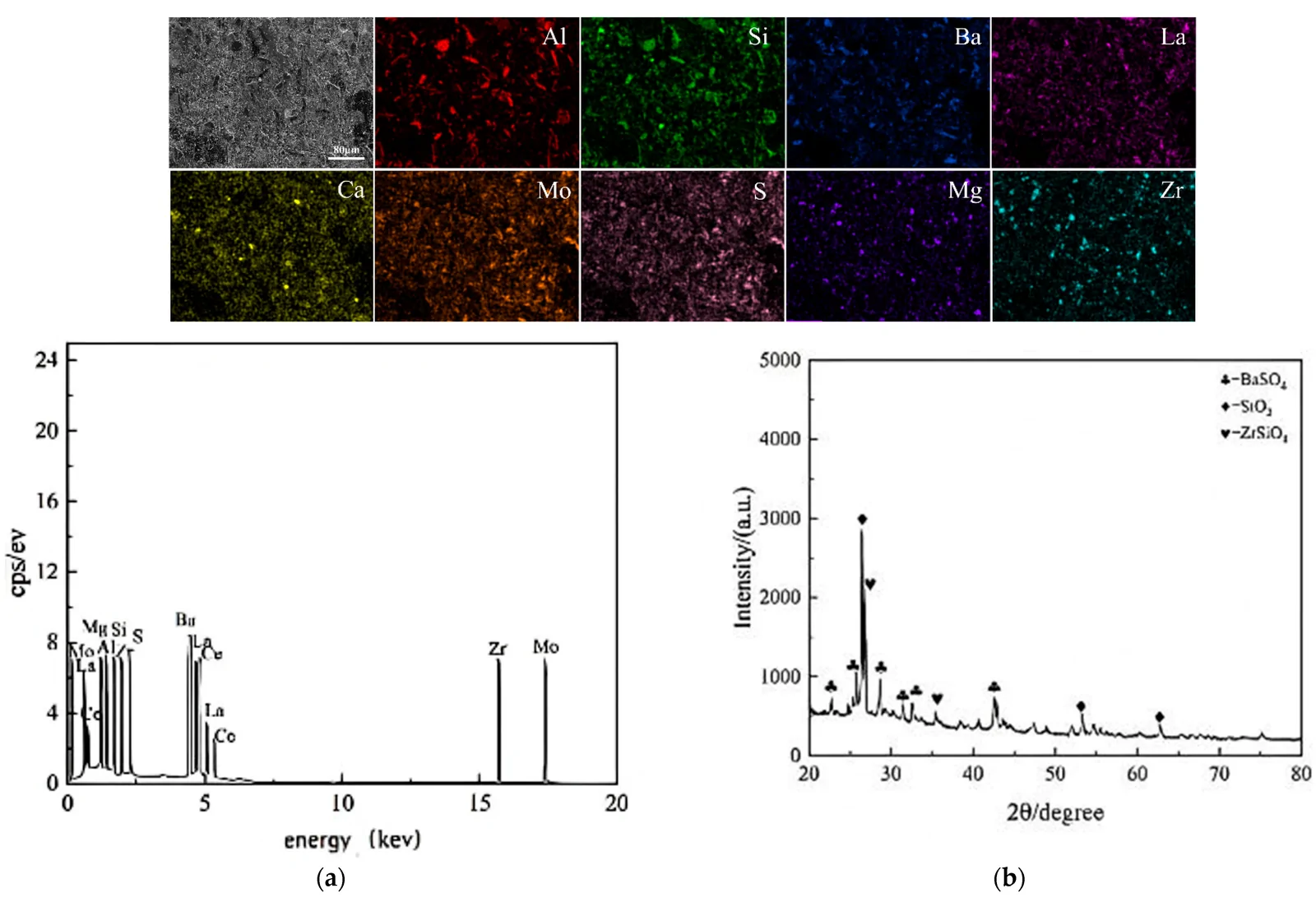

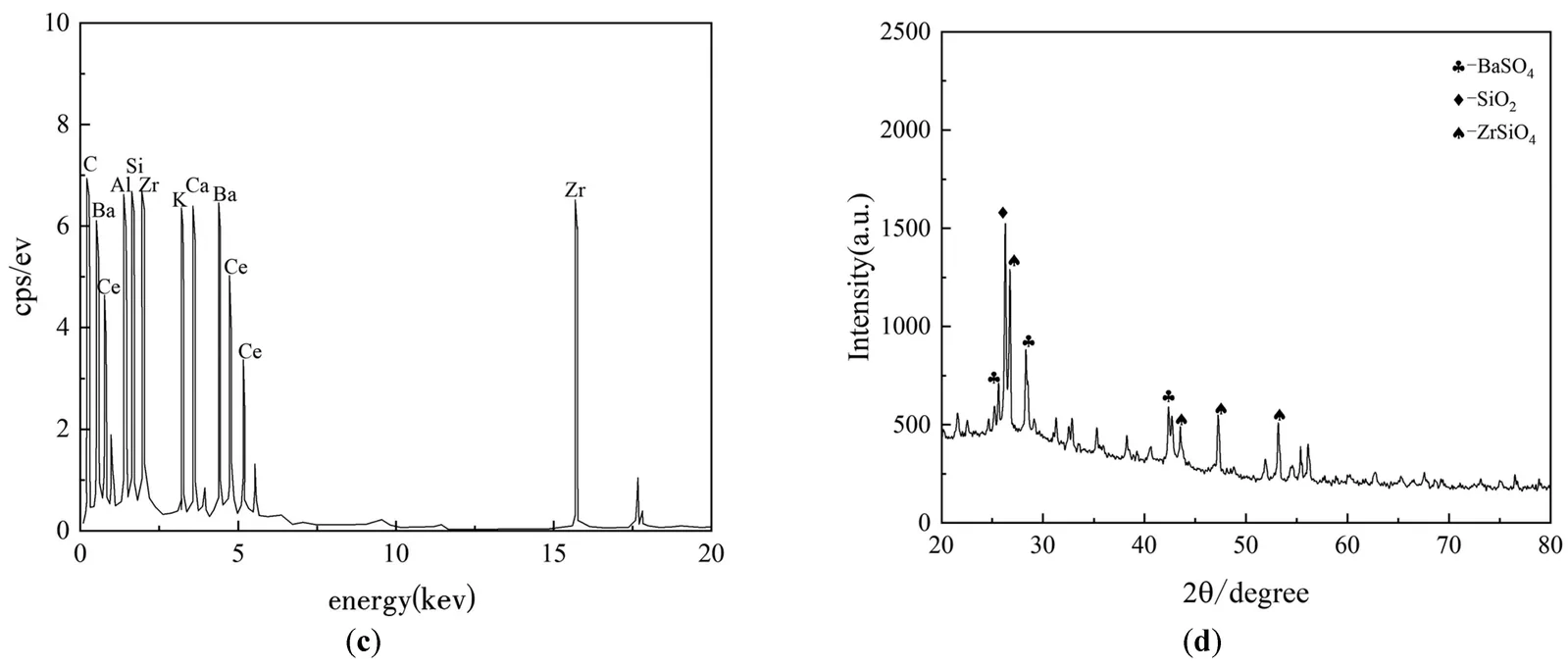
Comparative EDS elemental mapping and XRD patterns of the worn surfaces for (**a**) element peak of the optimal T3 specimen; (**b**) XRD diffraction pattern of the optimal T3 specimen; (**c**) element peak of the poor-performing T6 specimen and (**d**) XRD diffraction pattern the poor-performing T6 specimen.

The EDS elemental maps and XRD patterns for specimens T3 and T6 are presented in Figure 9.

The EDS analysis (Figure 9a,c) reveals a quantitative spatial distribution of key functional elements. For the optimal T3 specimen (Figure 9a), the signals for Mo, Si, and Al are uniformly distributed across the mapped area, with a coefficient of variation (CV) of pixel intensity below 15% for each element, indicating the formation of a homogeneous composite tribofilm. In contrast, the distribution on T6 (Figure 9c) is highly heterogeneous, suggesting ineffective integration of fillers and a patchy, unstable tribofilm.

The XRD phase analysis (Figure 9b,d) provides quantitative insights into the crystallinity and phase composition of the tribofilm. T3 exhibits sharp, high-intensity diffraction peaks for SiO_2_ and ZrSiO_4_, indicating the presence of highly crystalline, stable hard phases that contribute to mechanical integrity. The absence of distinct MoS_2_ peaks in T3 suggests its highly dispersed or amorphous state within the tribofilm, conducive to lubrication. For T6, alongside weaker crystalline peaks, the detectable but broadened MoS_2_ peak indicates partial agglomeration and poor exfoliation, correlating with its inferior lubricating function and higher wear.

### 3.6. Wear Mechanism Analysis

While raw-material micrographs are not presented, the morphological and crystallographic attributes of the fillers (spherical FAHMs, lamellar talc, layered MoS_2_) are well established in the literature and supplier datasheets. Moreover, the tribological evolution, directly captured by post-wear surface analyses (Figure 7, Figure 8 and Figure 9), provides robust indirect evidence for the effective dispersion and participation of the original fillers: the uniform elemental distribution (Figure 9a) and stable crystalline phases (Figure 9b) indicate an adequately homogeneous starting microstructure. Therefore, the following discussion of synergistic mechanisms, based on integrated performance data and post-wear microstructural evidence, reliably correlates the observed wear behavior with the known intrinsic properties of the raw materials. The effective formation of a uniform tribofilm in T3, for example, can be attributed both to standardized processing that promotes initial filler distribution and to internal coordination during sliding, without requiring separate micrographs of the pristine composite.

**Figure 10:**
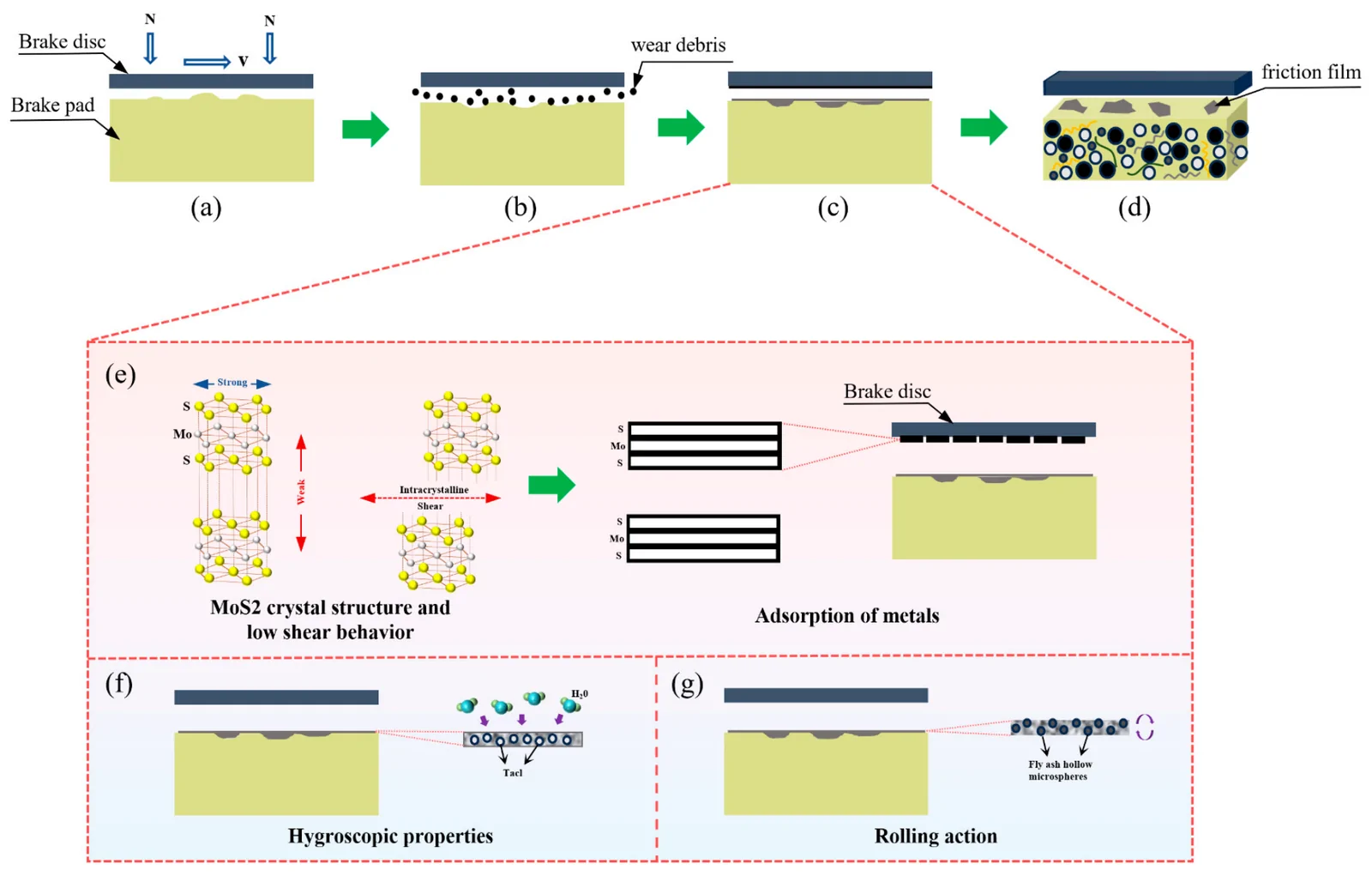
Wear mechanism diagram: (**a**) the brake disc and the brake pad interact with each other; (**b**) the formation of wear dbris; (**c**) the coordinated action of FAHMs, talc, and MoS_2_; (**d**) the formation of friction film; (**e**) MoS_2_ crystal structure and adsorption; (**f**) Tacl’s hygroscopic properties; (**g**) rolling action of FAHMS.

The wear process can be delineated into three consecutive stages (Figure 10). In Stage I (Figure 10a,b), surface asperities and hard filler particles interact with the counterface, generating fine debris through direct abrasive ploughing and micro-cutting. In Stage II (Figure 10b,c), this debris is progressively compacted and mixed with lamellar fillers (MoS_2_ and talc) under normal pressure and shear, forming an incipient third-body layer. The effectiveness of this transition determines whether the system enters a protective tribofilm regime or remains in a direct wear regime. In Stage III (Figure 10c), a continuous, adherent tribofilm stabilizes, accommodating interfacial shear via intrafilm sliding so that material loss occurs primarily through gradual film thinning, localized delamination, and re-compaction, rather than direct asperity penetration.

In the optimal T3 formulation, the coordinated action of FAHMs, talc, and MoS_2_ ensures rapid and stable transition to Stage III, with the tribofilm maintaining its integrity across the tested temperature range. In contrast, poorly formulated specimens such as T6 fail to reach or sustain this stage, reverting to direct wear characterized by deep grooving, extensive spalling, and high wear rates.

Each filler contributes distinct functions to this process. MoS_2_ acts as a solid lubricant and shear-plane provider [42]; its layered structure facilitates easy interlayer sliding, reducing the shear stress required for tribofilm formation and stabilizing the friction coefficient (Figure 4). In T3, the homogeneous distribution of Mo (EDS mapping, Figure 9a) confirms that MoS_2_ is effectively incorporated into the tribofilm, supplying lubricating nanosheets that prevent direct metal-to-composite contact and mitigate adhesive wear. the role of MoO_3_, formed through oxidation, is context-dependent: it can provide easy shear planes and improve lubrication [38,39], but excessive oxidation or abrasive morphologies may increase friction and wear. Thus, T3’s superior performance likely stems from maintaining the MoS_2_/MoO_3_ balance within an optimal range via the synergistic action of FAHMs and talc, rather than from completely suppressing MoO_3_ formation; however, this interpretation remains preliminary without XPS analysis. Talc serves as an interfacial conditioner and compressive reinforcer [43]; its platy morphology interlocks within the matrix, enhancing compressive strength (Figure 3) and promoting uniform pressure distribution, thereby sustaining tribofilm continuity under cyclic loading (Figure 4). FAHMs function as hard-phase reinforcements and micro-rollers [32]; their spherical shape and high hardness improve macro-hardness and shear strength (Figure 3), while acting as load-bearing sites that reduce plastic deformation and facilitate debris rolling, promoting compaction into a denser tribofilm [42].

These contributions are not merely additive but synergistic: FAHMs accelerate debris rolling and compaction, talc improves tribofilm cohesion, and MoS_2_ provides easy-shear planes that accommodate interfacial strain and prevent gross film fracture. This coordination culminates in a uniform, adherent, and thermally stable tribofilm (Figure 8c), which effectively separates the contacting surfaces, minimizes abrasive grooving, and reduces the wear rate to 0.7 × 10^−7^ cm^3^/(N·m) at 350 °C (Figure 6).

The wear mechanism in the developed composites is primarily abrasive-dominated, with adhesive components appearing in poorly formulated specimens. The optimized T3 system successfully transitions abrasive wear into a protective third-body tribofilm regime through the coordinated roles of FAHMs (hardness/shear strength), talc (compressive/interface stability), and MoS_2_ (lubrication/friction modulation). This mechanistic understanding not only explains the superior performance of T3 but also provides a design rationale for eco-friendly friction materials that avoid toxic antimony-based additives.

## 4. Conclusions

This study developed an eco-friendly ternary filler system to replace toxic antimony-based additives in resin-based friction materials. The L_9_(3^4^) orthogonal design identified the optimal formulation T3 exhibited excellent comprehensive performance. The T3 formulation meets the key performance benchmarks for wind turbine brake applications, including a stable friction coefficient within the 0.30–0.45 range, compressive strength exceeding 90 MPa, and a wear rate competitive with or superior to existing commercial and literature-reported systems. The core findings regarding filler functions and their coordination are synthesized as follows:

The combination of talc and FAHMs, as a structural filler package, collectively enhances the mechanical properties of the composite. The plate-like talc and spherical FAHMs complement each other: talc facilitates stress distribution and crack deflection through its layered interlocking, while FAHMs provide rigid load-bearing support and increase shear resistance. Together, they contribute to improved hardness, compressive strength, and shear strength, although their individual statistical weights cannot be separated in the current screening design.

The superior performance of the T3 formulation arises from a complementary functional coordination, not merely the sum of individual effects. This coordination manifests in the formation of a uniform, adherent, and stable tribofilm, where the FAHMs establish a robust mechanical skeleton, the talc plates reinforce the matrix structure, and the MoS_2_ laminates provide easy-shear planes, collectively ensuring the development of a protective layer that minimizes direct asperity contact, mitigates adhesive wear, and maintains consistent friction for low wear and high thermal recovery.

The T3 formulation successfully eliminates antimony compounds, thereby removing a source of heavy metal pollution. Simultaneously, it valorizes industrial solid waste (fly ash). This work demonstrates that high tribological performance, comparable to conventional systems, can be achieved through this eco-friendly, coordinated design strategy. The results provide a validated foundation for developing sustainable brake materials, particularly for applications like wind turbine braking where environmental and performance criteria are stringent. While the current study successfully identifies a promising eco-friendly formulation and elucidates its wear mechanisms, the long-term durability under real-world conditions, including environmental exposure (temperature/humidity cycles, UV radiation) and repeated braking-induced fatigue, remains to be evaluated. Future work will employ extended wear tests, accelerated ageing, and fatigue testing to fully validate the practical reliability of this material for wind turbine brake pads.

## Figures and Tables

**Table 1:** Summary of constituent materials used in the composite formulation, including grade/size, commercial source, and primary role.

Material	Specification/Mesh/Size	Supplier/Manufacturer	Primary Role
MoS_2_	1200 mesh	Hubei Bakro Petrochemical Co., Ltd, Xiaogan, China	Key variable filler
Talc	800 mesh	Tianjin Huasheng Chemical Reagent Co., Ltd, Tianjin, China	Key variable filler
FAHMs	100 mesh	Hebei Jingye Iron and Steel Co., Ltd, Shijiazhuang, China	Key variable filler
Phenolic resin	200–250 mesh	Nantong Sumitomo Bakelite Co., Ltd, Nantong, China	Binder
Aramid fibers	Length: 1.13 mm	Teijin Group, Tokyo, Japan	Reinforcing fiber
Ceramic fiber	Diameter: 1–15 μm	Jiangsu Ruike High-tech Material Co., Ltd, Huaian, China	Reinforcing fiber
Potassium hexatitanate	Diameter: 0.4 μm	Shanghai Kaijunfeng Industry Co., Ltd, Shanghai, China	Whisker reinforcement
Calcium sulfate whisker	Aspect ratio: 40–60	Zhengzhou Bocelli Ecological Engineering Co., Ltd, Zhengzhou, China	Whisker reinforcement
La_2_O_3_	10–20 μm	Ganzhou Liantuo New Materials Co., Ltd, Ganzhou, China	Thermal stabilizer
CeO_2_	10–20 μm	Ganzhou Liantuo New Materials Co., Ltd, Ganzhou, China	Thermal stabilizer
ZrSiO_4_	400 mesh	Chongqing Jinjuyuan New Material Technology Co., Ltd, Chongqing, China	Thermal stabilizer
BaSO_4_	1250 mesh	Wuxi Mesd Chemical Products Co., Ltd, Wuxi, China	Density modifier
Ca(OH)_2_	170 mesh	Tianxuan Composites (Shanghai) Co., Ltd, Shanghai, China	pH regulator
Natural flake graphite	—	Shenzhen Hanhui Graphite Co., Ltd, Shenzhen, China	Solid lubricant

**Table 2:** Orthogonal test table.

Specimen Number	Mass Fraction ω/wt%		
	Molybdenum Disulfide	Talc	FAHMs
T1	6	3	2
T2	6	5	3
T3	6	6	4
T4	8	3	2
T5	8	5	3
T6	8	6	4
T7	10	3	2
T8	10	5	3
T9	10	6	4

## Data Availability

The original contributions presented in this study are included in the article. Further inquiries can be directed to the corresponding author.
